# Pestilence and famine: Continuing down the vicious cycle with COVID-19

**DOI:** 10.1371/journal.ppat.1010810

**Published:** 2022-10-06

**Authors:** Sudipta Hyder, Rethy K. Chhem, Filip Claes, Erik Albert Karlsson

**Affiliations:** 1 Virology Unit, Institute of Pasteur du Cambodge, Phnom Penh, Cambodia; 2 Technology and Humanities, CamTech, Phnom Penh, Cambodia; 3 Hub of One Health, University of Puthisastra, Phnom Penh, Cambodia; 4 Emergency Center for Transboundary Animal Diseases (ECTAD) of the Food and Agriculture Organization of the United Nations, Regional Office for Asia and the Pacific, Bangkok, Thailand; 5 CANARIES: Consortium of Animal Market Networks to Assess Risk of Emerging Infectious Diseases through Enhanced Surveillance; University of Arizona, UNITED STATES

## Abstract

Despite the fact that we produce enough food to feed everyone on Earth, world hunger is on the rise. On the other side of the table, the obesity crisis also weighs heavily. Malnutrition is less about food than about socioeconomic factors such as conflict, poverty, and global disasters such as climate change and the novel Coronavirus Disease 2019 (COVID-19) pandemic. Nutrition and infectious disease exist in an intricate dance. Adequate and balanced nutrition is critical for appropriate response to infection and any changes in the balance can serve as a tipping point for the next pandemic. On the other hand, pandemics, such as COVID-19, lead to greater malnutrition. Both over- and undernutrition increase severity of disease, alter vaccine effectiveness, and potentially create conditions for viral mutation and adaptation—further driving the disease and famine vicious cycle. These long-term health and socioeconomic repercussions have direct effects at individual and global levels and lead to long-term consequences. Therefore, investing in and strengthening public health, pandemic prevention, and nutrition programs become vital at a much more complex systems level.

## Out of the frying pan and into the fire…

While many have bemoaned the past 2 years of the Coronavirus Disease 2019 (COVID-19) pandemic, there have been much worse times to be alive. Around 536 AD, a volcano erupted—possibly in Iceland—spilling millions of tons of ash into the atmosphere across the Northern Hemisphere [1]. The sun disappeared for 18 months. Crops failed. Millions starved. It also likely sparked the Plague of Justinian (the first documented bubonic or “Black” plague), leading to more famine and devastation. It happened again in the 1300s. A volcanic eruption somewhere in New Zealand led to years of cool, rainy weather preventing crops from ripening in Europe [2]. Waves of respiratory diseases further weakened the population, leading to greater famine. This Great Famine possibly primed an undernourished, weakened population for the Black Death to ravage through Europe in 1346, killing more than a third of the population [3]. In 1815, Mount Tambora erupted in Indonesia, causing “the year without summer” and, as a long-term consequence, the Irish Potato Famine of 1845, which led to mass starvation and many death from communicable diseases [4].

Are we detecting commonality here? The historical and biological Butterfly Effect of disease during a famine (or famine during outbreak) is an intricate dance. Infectious disease and malnutrition amplify bidirectionally. Catastrophes such as natural disasters, climate change, war, and economic collapse lead to starvation and infection. Weakened immune systems reduce resistance to further infection, and, ultimately, more malnutrition in a vicious cycle with long-lasting impacts on public health [5].

**Fig 1:**
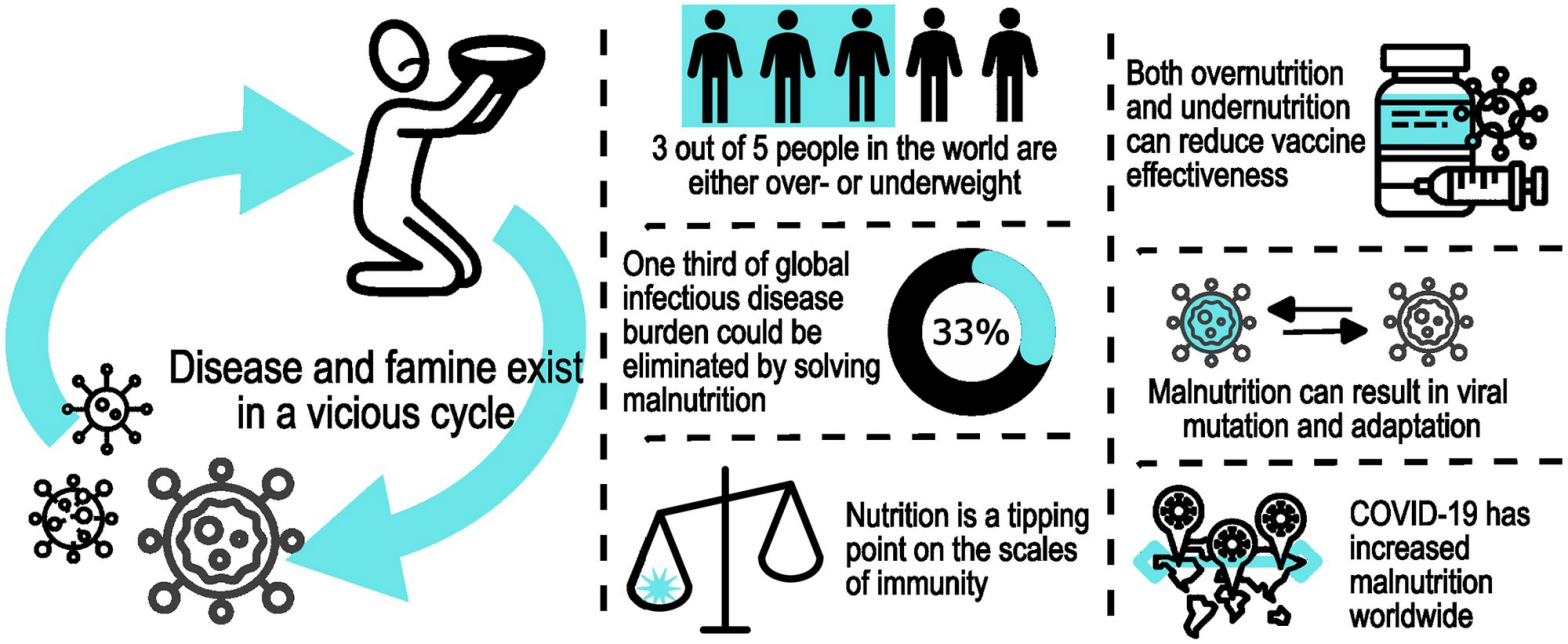
Some key facts and points on the vicious cycle of nutrition and infectious disease. Nutrition is critical for immune response and protection from infection. A significant proportion of people in the world are malnourished, a major concern for infectious disease. COVID-19 has increased malnutrition worldwide, creating conditions that may lead to the next pandemic. “People pack” by Dumitriu Robert, “Coronavirus pack” by Graphic Mall, “Coronavirus icon pack” by SkyClick, and “Coronavirus COVID-19 pack” by Eucalyp Studio are all available at http://www.iconfinder.com under a Creative Commons (Attribution 3.0 Unported) license (https://creativecommons.org/licenses/by/3.0/). “Phosphor Thin Vol.4 pack” by Phosphor Icons is available at https://www.iconfinder.com/iconsets/phosphor-thin-vol-4 under a Creative Commons Attribution 4.0 International license (https://creativecommons.org/licenses/by/4.0/).

While the COVID-19 pandemic may not directly involve a volcano, history repeats itself. Disease leads to increased poverty, hunger, and changes in socioeconomic behaviors. Thus, insights from past pandemics frame the current COVID-19 hunger crisis and can inform actions to mitigate negative consequences from COVID-19 and future disease outbreaks. One of the most lasting legacies of the pandemic will be increased malnutrition—both undernutrition and obesity (Fig 1). These scars will echo with resounding implications, not only for pandemic recovery, but also for the future of emerging and endemic infectious diseases. Several factors should be considered to reflect on the future of nutrition and infectious disease through the lens of COVID-19:

1. **Hunger: What doesn’t kill you, just makes you more vulnerable to something else killing you**
  Every year, over 5.5 million children die before the age of 5, mostly in Africa and Asia [6]. Half of these children are undernourished. Why? Apart from the necessity of food for sustaining life, nutritional deficiencies decrease immune response, resulting in increased susceptibility, severity, and mortality from diseases. Indeed, about 32% of the global infectious disease burden could be alleviated by eliminating malnutrition [7]. Undernourished children are more susceptible to respiratory infections, diarrhea, measles, and malaria, to name a few [7]. Unfortunately, infection also leads to poor nutrition by causing anorexia and malabsorption and increasing nutrition requirements for immune cells. This leads to even more undernutrition, increasing susceptibility to disease, and moving further down the vicious cycle [8,9].
2. **The weight of obesity in infectious disease**
  Malnutrition isn’t just about not having enough food, it’s also about eating too much. Obesity impairs immune function, leading to increased susceptibility to infection with a number of different pathogens including influenza and COVID-19 [10]. Why? Because your fat also talks to your immune system. Obesity alters adipose tissue signaling, resulting in miscommunication with the immune response and compromising function to deal with infections [11]. Therefore, this interrelationship between adipose tissue, immune response, and infections can exacerbate infection.
3. **You are what your mother and mother’s mother ate**
  There is a direct linkage between maternal malnutrition and the nutritional status of her child. Early malnutrition significantly increases the risk of developing chronic diseases in adults, resulting in a weakened immune response to infectious diseases, and, beyond the age of 2 to 3 years, the effects of chronic malnutrition become almost irreversible [12,13]. Children of women who lived through the Hungry Winter of 1944 to 1945 in the Netherlands and the Chinese Great Famine of 1959 to 1961 faced higher rates of obesity, increased risk for hypertension and cardiovascular disease, and increased rates of diabetes [12–14]. Even the grandchildren of these women were smaller than average and had a higher chance of becoming obese during adulthood [14]. But we shouldn’t point fingers at moms just yet. The nutritional environment both before conception and throughout gestation influences adult metabolism, but they are just one factor that involved in development.
4. **Vaccination efficacy is like a bowl of porridge**
  While opinions on vaccination are undoubtedly mixed, we cannot deny vaccines have greatly improved public health. Smallpox was eradicated in 1979. Polio, measles, and diphtheria are becoming rare. However, vaccine efficacy requires a functional immune system and nutrition is key to bolstering the immune response. Indeed, nutrition is a tipping point on the scales of immunity. Like Goldilocks, effective immunity requires everything to be just right. Too hot or too cold? Things start to break down. Undernutrition has been found to play a role in altering vaccine response for tuberculosis [15], rotavirus [16], measles, and pneumococcus [8]. Obesity also interferes with the ability to mount an effective immune response to vaccination. Obesity also reduces antibody response to hepatitis B and tetanus vaccination. While some associate this reduced response with improper needle length or vaccination technique, it’s also about the antibodies [17]. Compared with healthy-weight adults, vaccinated obese adults are at twice the risk of influenza-like illness or influenza despite equivalent serological response to the influenza vaccination [18].
5. **Sometimes, viruses are what you eat**
  Nutritional excess or deficiency both dampen the host immune response and alter cellular metabolism, which creates an advantageous environment for viruses to explore the sequence space and potentially emerge with advantageous mutations [19]. Take obesity. The altered microenvironment associated with obesity supports a more diverse viral quasispecies—a mutant cloud of genomes that collectively infect, replicate, and spread among hosts—potentially evolving pathogenic variants capable of inducing greater transmission or disease severity [20]. On the flip side, nutritional deficiencies also change the genetic composition of viruses. In selenium-deficient and vitamin E-deficient mice, genetic mutations arise in coxsackie B and influenza virus populations, and benign strains become more virulent, even to well-nourished hosts [20].

## So, what does this all mean? Nutrition and infection in the “New Normal”

Before the pandemic, the world was already malnourished. Only 2 out of every 5 people on the planet were considered healthy, and many of those probably had a vitamin deficiency [6]. Despite decades of effort, 820 million people in the world still faced hunger shortages and chronic malnutrition at the end of 2019 [21]. Economic slowdowns and downturns already undermined food security [22]. Then along came a pandemic. Lockdowns, supply chain issues, mass job losses, and political crises exacerbated major socioeconomic challenges and furthered food scarcity and inaccessibility. In all, it is estimated that COVID-19 has threatened access to a healthy diet for nearly 2 billion people [21], 6.7 million of those being children under the age of 5 [23].

Along with hunger, global obesity had nearly tripled since 1975, with almost 40% of adults being overweight in 2016 [24]. During the pandemic, as some were edging closer to wasting and stunting, others were ballooning further into obesity. Pandemic stress, job loss, decreased access to nutritious foods, and increasingly sedentary lifestyles have exacerbated the obesity epidemic [25]. For children in the United States, rate of body mass index increase nearly doubled during the COVID-19 pandemic [26]. As COVID-19 continues to aggravate malnutrition globally, it feeds the vicious cycle of malnutrition and infection with potentially even greater morbidity and mortality down the road. Furthermore, prophylactics for nutritionally compromised populations continue to remain uncharted territory, as a majority of vaccine seroconversion and effectiveness studies are performed on healthy individuals, a minority of the current global population [11].

So, even before the pandemic ends, we already need to start considering the next. The majority of critically malnourished people reside in global hotspots of emerging and endemic infectious diseases. Pathogens will continue spilling over. The vicious cycle will continue. Investing in and strengthening public health, pandemic prevention, equal access to healthy food, and good nutritional programs is vital. Even combating climate change is critical for agriculture, disease exposure, and, yes, volcanoes. About 1,500 potentially active volcanoes exist around the world. As our planet warms, these volcanoes are projected to become more active and the cooling effects of their eruptions are expected to increase by 15%, massively increasing repercussions. No one wants another worst year to be alive.
